# Refractory Sporotrichosis Caused by *Sporothrix globosa*

**DOI:** 10.4269/ajtmh.22-0666

**Published:** 2023-03-20

**Authors:** Xiujiao Xia, Ze-Hu Liu, Hong Shen

**Affiliations:** Department of Dermatology, Hangzhou Third People’s Hospital, Affiliated Hangzhou Dermatology Hospital, Zhejiang University School of Medicine, Hangzhou, China

A 49-year-old woman presented to our dermatology clinic with a 7-month history of progressively increasing nodules over the right upper arm. The patient worked as a farmer in eastern China and noted that the nodules had developed after accidental scratches to the arm from a piece of wood. Physical examination revealed erythematous–purpuric nodules with a suppurative tendency following the ascending lymphatic path of the right upper arm (Figure 1A). The results of routine laboratory tests were within normal ranges. Histopathology showed multinucleated giant cells containing round spores in periodic acid–Schiff stain (Figure 1B). The tissue fluid and tissue culture on Sabouraud dextrose agar at 25 °C for 10 days revealed colonies of *Sporothrix* spp. The isolate was identified as *Sporothrix globosa* by ITS sequencing (GenBank accession no. ON311113).

**Figure 1.**
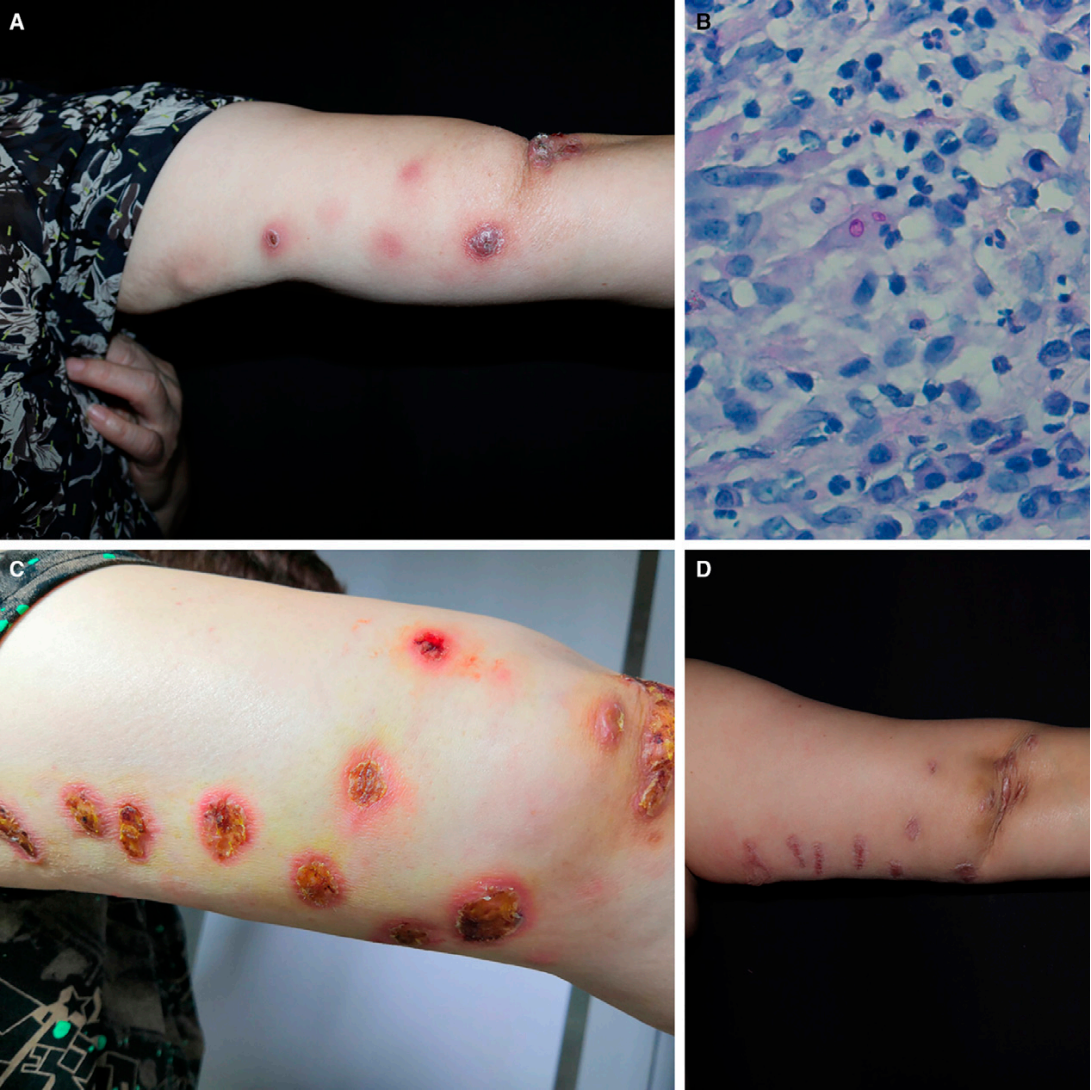
(**A**) Clinical appearance of the skin lesions before treatment. (**B**) Histopathology showing round spores in multinucleated giant cells (periodic acid–Schiff ×400). (**C**) Appearance of the skin lesions after 5 months of treatment with itraconazole 100 mg/day. (**D**) Complete regression of lesions after 11.5 months of treatment.

Treatment was started with itraconazole 200 mg daily. After 6 weeks of treatment, there was improvement. Considering the risk of drug-induced liver injury, the patient reduced the dose to 100 mg/day without authorization. Five months after the treatment, no improvement was seen, and new lesions developed. The patient was again admitted to our outpatient clinic. Physical examination showed multiple plaques (Figure 1C). Blood biochemical evaluation revealed no abnormalities. Direct examination (fluorescent staining) of the tissue fluid from the lesions showed nonspecific germinal tube and hyphae. Fungal culture of the tissue fluid was positive. Therapy was changed to itraconazole 400 mg/day, as well as regular liver function monitoring. After another 5 months of treatment, the skin lesions completely resolved, leaving slight residual atrophy in the areas of previous skin lesions. Five months after the end of treatment, there was no recurrence of disease (Figure 1D).

The Infectious Diseases Society of America guideline recommends itraconazole 200 mg/day continuous regimen as the first choice to treat cutaneous sporotrichosis.^1^ The course of treatment usually ranges from 3 to 6 months. In previous studies, only 6% of patients needed to increase the dose of itraconazole from 100 mg/d to a higher dose to achieve sporotrichosis cure, and only 1.2% needed to switch to other drugs, such as terbinafine or potassium iodide.^2^ This patient’s clinical response to itraconazole therapy (200 mg/day) was initially satisfactory. However, after 45 days on itraconazole, the patient reduced the dose to 100 mg/day without authorization, the lesions gradually worsened. This case suggests that it is important for clinicians to provide close monitoring during the treatment of sporotrichosis. Therapeutic drug monitoring establishes clinically useful threshold values for clinical outcomes and adverse events.^3^ These measures can help clinicians adjust a therapeutic regimen over time. In addition, measurements of antifungal concentrations could guide dosage to achieve adequate pharmacodynamics (PD) target. A trough level range of 0.5 to 1 mg/L is generally used as PD target of itraconazole.^4^

## Financial Disclosure

This work was supported by the Hangzhou Science and Technology Bureau, China (Grant no. 202004A17).
